# Racial and Ethnic Disparities in Fertility Preservation for Women with Cancer in the United States—Identifying Systemic Barriers and Proposing Solutions Through the DART Hypothesis

**DOI:** 10.3390/cancers18050828

**Published:** 2026-03-04

**Authors:** Jasmin Mahabamunuge, David B. Seifer

**Affiliations:** 1 Division of Reproductive Endocrinology and Infertility, Department of Obstetrics and Gynecology, Women and Infants Hospital and Warren Alpert Medical School of Brown University, Providence, RI 02903, USA; 2 Department of Obstetrics, Gynecology, and Reproductive Sciences, Yale University School of Medicine, 333 Cedar Street, New Haven, CT 06510, USA; david.seifer@yale.edu

**Keywords:** fertility preservation, cancers in women of reproductive age, chemotherapy, race, ethnicity, disparities, DART hypothesis, bias, assisted reproductive technology, in vitro fertilization, access, treatment outcomes, public health

## Abstract

Continuing advances in cancer diagnosis and treatment, particularly in reproductive-aged patients, has led to numerous national medical organizations, including the American Society for Reproductive Medicine (ASRM), the American College of Obstetrics and Gynecology (ACOG), and the American Society for Clinical Oncology (ASCO), recommending prompt discussion of the potential gonadotoxic effects of chemotherapy and referral to a fertility specialist for counseling regarding possible fertility preservation. Despite overall increased utilization of assisted reproductive technology (ART) in the United States (US), racial and ethnic disparities persist on a multisystem level, ranging from decreased access and utilization of ART to inconsistent and delayed counseling to worse outcomes. While innovations in ART and cancer treatment continue to evolve in parallel, the beneficial impacts have been disparate and more limited in minority populations. This review specifically highlights racial and ethnic disparities in fertility preservation for women with cancer in the United States, highlights the underdeveloped state of this literature, and identifies possible pathways for improvement using the Disparities in Assisted Reproductive Technology (DART) hypothesis as a template. We address three main bottlenecks resulting in delay from time of cancer diagnosis to utilization of fertility preservation services.

## Simple Summary

This narrative review article focuses on disparities in fertility preservation of women from racial and ethnic minority groups diagnosed with cancer. We discuss the multiple bottlenecks and delays that occur from the time of diagnosis, and initial counseling, to surgical treatment, to referral to a fertility specialist, and finally to access/utilization of fertility preservation services. We also suggest possible solutions to bridge these gaps using the DART hypothesis as a template.

## 1. Introduction

Advances in cancer diagnosis and treatment, particularly in young adults and adolescents, have resulted in improved overall cancer survival rates [1]. This has shifted expectations for provider counseling at the time of diagnosis to include possible life outcomes within the context of survivorship. One such example is future fertility. Cancer and its treatment can adversely affect fertility via multiple modalities, including the presence and progression of underlying disease, gonadotoxic effects of chemotherapy, surgical excision of reproductive organs, and radiation [2]. These realities are seldom a surprise to patients and are often on their minds from the time of initial diagnosis. A 2018 systematic review found that fertility-related psychological distress is prevalent among people diagnosed with cancer and persists from the time of diagnosis through treatment and survivorship [3]. Consequently, the American Society for Reproductive Medicine (ASRM), the American College of Obstetrics and Gynecology (ACOG), and the American Society for Clinical Oncology (ASCO) recommend promptly discussing the effects of known or potential gonadotoxic therapies, and surgical interventions, on fertility for reproductive-aged patients prior to starting treatment [4,5,6]. Additionally, they advise for immediate referral to a reproductive endocrinology and infertility (REI) specialist for evaluation and consideration of treatment for patients who desire or are uncertain about fertility preservation (FP).

While national efforts are underway to ensure reproductive-aged patients with a new cancer diagnosis are connected to an appropriate specialist for counseling and possible FP, studies suggest this practice has not yet been universally adopted [7,8]. Additionally, emerging evidence unfortunately demonstrates disparities in FP for cancer patients based on sociodemographic factors including race/ethnicity, geographic location, and insurance status [9,10,11,12]. Though the presence of disparities in FP is not entirely surprising given the well-established disparities in fertility and cancer-related treatment independently, the intersection of these disparities is an additional cause for concern given their potential compounding effects. As such, disparities in FP warrant additional exploration and troubleshooting.

## 2. Methods

Though this was not a systematic review, we utilized a structured approach for a literature review to facilitate reproducibility and transparency. A medical literature search was performed in PubMed. Phrases used in the search included “fertility preservation” AND “disparities” AND “cancer.” Our search period spanned 2005–2025. Seventy-eight publications were initially found. Articles were then assessed for relevance to FP, specifically for patients with cancer planning or considering FP prior to chemotherapy, and for relevance to racial/ethnic disparities by including articles that reported race/ethnicity as an outcome and/or main discussion topic. We included multiple publication types in this review, however prioritized peer-reviewed clinical research studies given the limited number of investigations relevant to this topic.

This narrative review aims to provide a focused summary of racial and ethnic disparities in FP for women of reproductive age with cancer in the United States and provide a framework for understanding and addressing such disparities. While this article uses the term “woman” to describe people born with a uterus and ovaries, we recognize and acknowledge that not all people born with these reproductive organs identify as a woman. In addition to reviewing the current landscape of these inequities, we highlight the limited number of studies that have rigorously investigated this topic and call for additional research. Furthermore, we identify three major bottlenecks that contribute to and perpetuate these disparities and comment on the applicability of the Disparities in Assisted Reproductive Technology (DART) hypothesis to bridge these disparities. The DART hypothesis is a robust framework previously established and employed to not only understand but also to improve disparities in fertility access, treatment, and outcomes [13,14,15]. We believe the DART hypothesis has potential to serve as a template in providing possible mitigating solutions to improve care for patients existing at this intersection of disparities in oncological health care.

## 3. Fertility Preservation Overview

FP consists of several interventions aimed at preserving reproductive autonomy in preparation for the adverse reproductive effects of cancer treatment, including diminished ovarian reserve, early menopause, menstrual cycle dysfunction, and infertility. In women, the main options for FP consist of oocyte cryopreservation, ovarian tissue cryopreservation, ovarian transposition, and gonadotropin-releasing hormone agonists (GnRHas) [16]. Temporality is crucial, as nearly all of these interventions occur *prior* to initiating cancer treatment. However, GnRHas are administered concomitantly with gametotoxic chemotherapies for ovarian suppression when cancer treatment cannot be delayed or when time does not allow for any of the previously listed interventions prior to beginning chemotherapy [2,17,18,19]. While GnRHas are not typically a first-line intervention for FP, emerging data reveal time to return of menses can be predicted in premenopausal breast cancer patients undergoing simultaneous GnRHa and tamoxifen therapy depending on the GnRHa formulation/duration of treatment, thus potentially predictably preserving some reproductive function [20].

Since 2012, when the ASRM no longer considered oocyte cryopreservation to be experimental, utilization of oocyte cryopreservation has substantially and consistently increased. However, a 2021 large retrospective cohort analysis of 29,631 oocyte cryopreservation cycles from 2012 to 2016 using the Society for Assisted Reproductive Technology Clinical Outcome Reporting System (SART CORS) found that oocyte cryopreservation is underutilized in minority groups [21,22]. This study by Katler et al. included all cycles, and therefore established that oocyte cryopreservation, regardless of indication, is underutilized in minority populations [22]. In a more recent 2025 study using SART CORS data to assess national trends in planned oocyte cryopreservation, subsequent oocyte utilization, and outcomes of oocyte warming cycles, planned oocyte cryopreservation was most common among patients identifying as White and Asian and far less common for racial and ethnic minorities [23]. Unfortunately, this discrepancy is ubiquitous, trickling down and impacting FP for patients with a cancer diagnosis as well.

## 4. Current Racial and Ethnic Disparities in Fertility Preservation for Women with Cancer in the US

Below, we systematically summarize racial and ethnic disparities in FP for women with cancer in the US. Disparities emerge at multiple time points starting at the time of initial diagnosis when the effects of cancer treatment on fertility are introduced and discussed through counseling, to surgical treatment, to referral to an REI specialist, to the point of accessing and/or utilizing FP services, thus creating serial identifiable bottlenecks leading to delayed or absent FP due to time constraints and urgency for treatment of the diagnosed cancer.

### 4.1. Fertility-Sparing Treatments

In a population-based cohort study of over 7700 patients 18–45 years old diagnosed with early cervical (stage IA, IB), endometrial (grade 1, stage IA, IB), or ovarian (stage IA, IC) cancer during 2000–2015, using linked data from the California Cancer Registry, the California Office of Statewide Health Planning and Development, and the SART CORS database, racial and ethnic differences were observed in fertility-sparing treatment and ART use. In this study, fertility-sparing treatment was defined as interventions that allowed for the retention of at least one ovary and the uterus. Additionally, ART was defined as one or more autologous embryo- or oocyte-freeze cycle or embryo transfer cycle after cancer diagnosis. Interestingly, fertility-sparing treatment was more likely among patients in racial and ethnic minority groups for cervical and endometrial cancers. However, of the few individuals who used ART in the study (0.6%–1.1% depending on cancer type), they were more likely to be non-Hispanic White. In fact, no non-Hispanic Black or American Indian patients with cervical, endometrial, or ovarian cancer used ART [10].

While there is a shortage of studies focused on sociodemographic disparities in fertility-sparing surgical treatments, the previously cited well-powered study draws attention to this important topic, as the absence of fertility-sparing surgical treatment can preclude the possibility of future fertility. Additionally, inconsistent guidelines may contribute to disparities in fertility-sparing surgical treatment. As Erden et al. noted in their narrative review, multiple different guidelines support fertility-sparing treatment for select patients with grade 1, stage IA endometrioid endometrial carcinoma, stage IA1–IB1 cervical tumors measuring less than 2 cm without high-risk features, borderline ovarian tumors, and most malignant germ cell tumors; however, recommendations for higher-stage disease and less common histologies are inconsistent [24]. Studies focused on further evaluating these cases, stratified by demographic characteristics, are necessary to understand how bias may influence outcomes in fertility-sparing treatment when guidelines become increasingly subject to individual interpretation.

### 4.2. Counseling

Patient counseling focused on the reproductive repercussions of cancer treatment is essential for reproductive-aged patients. Likewise, discussions informing patients of the options available for preserving future fertility are also essential. Patients themselves may not be aware of the reproductive effects of essential cancer therapies, but studies show they desire and expect to learn about this from their physician. In a survey study of young adult cancer survivors diagnosed with cancer between the ages of 15 and 35 years, 40% of non-White respondents reported an unmet need for infertility information compared to 28% of White respondents [25]. Additionally, studies show differences in patient understanding of the impacts of cancer treatments on fertility based on race/ethnicity. In a cross-sectional survey of African American women with early-onset breast cancer in Florida, 45.8% reported being aware of the potential impact of cancer treatment on fertility and 56.3% reported that their providers discussed fertility with them [26]. Notably, many studies investigating patient counseling rely on patient recall, which increases the risk of recall bias. Consequently, to investigate disparities in FP counseling while minimizing the impact of recall bias, a study by Lawson et al. reviewed electronic medical record data documenting FP counseling completed at a single academic institution [27]. The study found disparities in physician counseling based on race and ethnicity, with Black women having the lowest rate of documented FP counseling (62% White, 65% Asian, 53% Black, 62% of unknown race, and 83% (5/6) Hispanic women (*p* < 0.05)) [27]. More multisite and national-level studies are needed to corroborate these findings and assess their generalizability. Provider comfort and knowledge regarding FP options seems to contribute to this phenomenon. A cross-sectional study of FP referral patterns among oncologists found that despite 90% of oncologists stating they were “very knowledgeable “or “aware of” FP options, only 17% had experience with embryo cryopreservation [7]. Thus providers may be even less comfortable and familiar with FP options than they realize.

### 4.3. Timely Referral

While it is the responsibility of the diagnosing clinician to inform their patients of the potential gonadotoxic effects of cancer treatments such as chemotherapy and to allow patients the option to pursue FP when clinically able, they are not expected to do so alone. A collaborative approach via referral to an REI specialist is recommended by the ACOG, ASRM, and ASCO. However, as was the case with reproductive counseling, which is a prerequisite for offering referral to an REI, differential referral patterns exist based on race and ethnicity. In a retrospective cohort study of women aged 18–42 years diagnosed with new breast, gynecologic, hematologic, or gastrointestinal cancer at a single academic institution between 2008 and 2010, the odds of FP consultation were approximately two times higher for Caucasian women (OR 2.4; 95% CI 0.9–6.2) [28]. Importantly, this study underscores the overall low rates of FP consultation in eligible patients (20.6%). Concerningly, multiple studies, including a recent 2026 cross-sectional study, have found that far less than half of US physicians are following the guidelines from ASCO and routinely referring patient to an REI specialist [7,29,30]. There are currently no published investigations of time from cancer diagnosis to REI referral based on sociodemographic factors, such as race and ethnicity, in patients newly diagnosed with cancer seeking FP. However, racial and ethnic disparities have been demonstrated in an increased interval to REI specialist referral in the general infertility population, contributing to lower rates of ART utilization in racial and ethnic minorities. We suspect this may also be a contributing factor for access to FP services as well. However, dedicated investigations of this possible contributing factor are warranted.

### 4.4. Access and Utilization of Fertility Preservation

After patients from racial and ethnic minority groups with a newly diagnosed cancer have been counseled and referred to a REI specialist, another bottleneck occurs at the level of access and utilization of FP. In a 2012 study of 1041 patients diagnosed with leukemia, Hodgkin disease, non-Hodgkin lymphoma, breast cancer, or gastrointestinal cancer between ages 18 and 40, 61% of patients were counseled regarding the risk of cancer treatment on fertility by the oncology team, with no significant differences seen based on race/ethnicity. However, only 4% of the women in this study underwent FP, and of these women, none identified as Black. Additionally, Latina-identified women were 80% less likely to undergo FP than Caucasian women (OR 0.2, 95% CI 0.0–1.3). Though this difference did not reach statistical significance, possibly due to the overall low sample size of patients seeking FP, another study investigating sociodemographic differences in female patients accessing fertility services after a cancer diagnosis in a representative sample of the US population found that patients identifying as Hispanic (OR 0.32, CI 0.06–1.91) and non-Hispanic other (OR 0.18, CI 0.01–2.49) were less likely to utilize fertility services [31,32]. While these findings are compelling, larger studies including multisite and national studies are needed to understand if these findings can be extrapolated on a population level. Additionally, larger samples inclusive of different types of cancer may help to elucidate how cancer type may effect of FP utilization.

Partly in response to a secondary analysis by Lee et al. showing that neither race nor ethnicity is predictive of whether women will utilize FP services prior to cancer treatment at academic institutions, Voigt et al. assessed whether racial and ethnic disparities impact access to FP services via a different method [33,34]. They compared the racial composition of women accessing medically indicated FP services at a clinic in New York City with a racial composition representative of the proportion of reproductive-age women diagnosed with cancer in New York City. They found a statistically significant difference in the observed versus expected racial distribution of patients accessing medically indicated FP, with particularly low utilization in Black- and Hispanic-identified patients [33,34]. While this study was completed at a single institution, its main strength was having a truly representative study sample, as it pertained to race and ethnicity in a given region. Given this regional specificity, it is difficult to assess if these finds can be extrapolated to other areas in the US.

## 5. Pathways for Accelerated Change—DART Hypothesis Revisited

It has been well established that racial and ethnic disparities exist at the level of cancer diagnosis, treatment, and survival outcomes [35,36]. The same is true for racial and ethnic disparities in broader ART access, treatment, and reproductive outcomes, with minority populations having less favorable outcomes [37,38]. Many solutions to the racial and ethnic disparities in reproductive care have been suggested, including but not limited to increasing patient education, increasing the number and availability of fertility clinics, educating physicians about implicit bias, recruiting a more diverse physician workforce, more thorough completion of SART demographic data, insurance mandates, engaging stakeholders, etc. [15,39]. Many of these suggestions are valid and applicable when considering how racial and ethnic disparities can be improved for patients with a new cancer diagnosis seeking FP. However, by virtue of existing at the intersection of two independent types of disparities, this patient population has some unique needs. Additionally, a shift toward provider factors rather than patient factors may be seen in this population. For instance, patient factors like cultural stigma concerning infertility and the need for fertility assistance may be less prominent in the setting of cancer, while provider factors such as REI referral patterns may become even more influential. Therefore, a discussion of how to improve the uniquely unmet needs of this population is warranted. We suggest addressing this systematically using the Disparities in ART (DART) hypothesis as a template for opening existing bottlenecks and expediting current delays.

**Figure 1:**
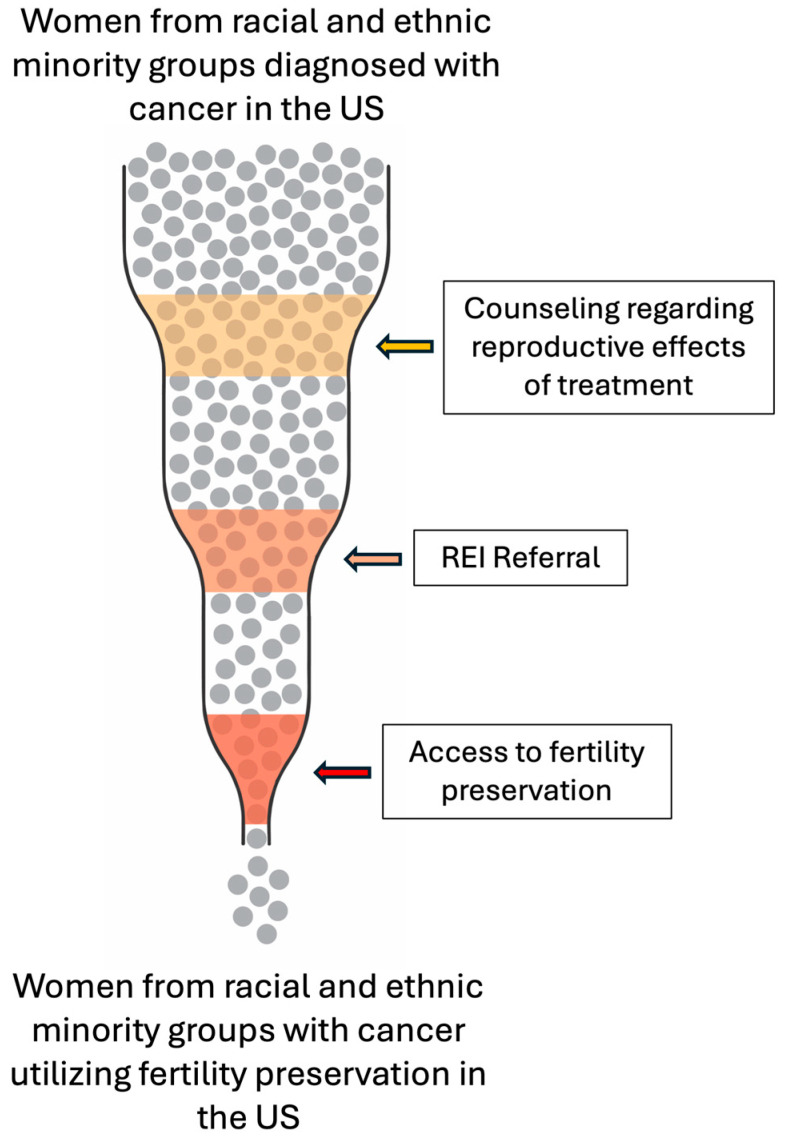
Depiction of the three main identifiable systemic bottlenecks contributing to racial and ethnic disparities in FP for women with cancer in the United States.

The DART hypothesis in racial and ethnic disparities in access and outcomes of IVF treatment in the US was initially proposed by Seifer et al., 2013 in the book chapter “Toward a better understanding of racial disparities in utilization and outcomes of IVF treatment in the USA,” and has been expanded and revisited in 2022, 2024, and 2025 [13,14,15,40]. This approach calls for identifying, integrating, and addressing the multiple factors contributing to racial/ethnic disparities in ART. We employ this method for addressing racial/ethnic disparities in FP for women with cancer in the US and specifically target the major bottlenecking events (Figure 1) that have been proven to contribute to this phenomenon.

As discussed above, one of the earliest factors contributing to disparate care for racial and ethnic minorities occurs at the time of diagnosis, when patients are under-counseled or not counseled at all regarding the reproductive consequences of cancer treatment. This should be considered an essential component of patient counseling. Additionally, just as careful documentation of the patient–provider conversation regarding diagnosis and prognosis is expected, documentation of the discussion of the reproductive effects of treatment should also be carefully documented. Previous studies have found that providers cite lack of education and knowledge regarding the reproductive effects of cancer treatment as a reason they forgo this [7,28,29,41]. A major risk of this lack of counseling is patients turning to unregulated online sources for information, which studies show are often inadequate and/or incorrect [42]. This underscores the pressing need for increased education regarding the reproductive effects of cancer treatment and basic options for FP in the curriculum for oncologists in training. For providers who are not currently in training, patient and physician informational resources, such as reading materials and decision aids, conveying accurate information show promise for bridging this gap [43,44]. Recent data from a study by Shliakhtsitsava et al. found that a web-based survivorship care plan providing information regarding evidence-based fertility management strategies to patients aged 18–40 diagnosed with breast cancer was effective at addressing patient concerns and increasing referrals and access to fertility specialists [45]. Specifically, this intervention revealed an improvement in pregnancy and fertility-potential concerns (RR = 3.5, 95% CI = 1.01–12.34, *p* = 0.05) and an increase in likeliness to receive a fertility specialist referral, schedule a fertility consult, or undergo fertility treatment (RR = 5.6, 95% CI = 1.28–24.73, *p* = 0.02) [45]. Patient educational interventions appear to be a highly effective tool for addressing disparate counseling.

Lastly, unintentional provider bias appears to play a role in discrepancies in counseling, as providers tend to provide FP counseling more often to patients who are married, highly educated, and/or to patients who bring this topic up on their own [27,32]. This highlights the need for and utility of universal provider bias training. Additionally, the timing of this counseling should be considered and conducted expeditiously, particularly for those 35 years and older, as advanced maternal age clearly impacts the prognosis for obtaining an optimal number and quality of cryopreserved oocytes and embryos and ultimately long-term reproductive outcomes following thawing [46].

Another important area where the opportunity for patients from racial and ethnic minority groups to pursue FP is lost is at the time of referral to an REI. Studies show academic centers have a lower burden of disparities in FP referrals and overall better service utilization [37,47]. This is partially due to the presence of subspeciality services that may be on-site, thus removing the barrier of travel, as well as established referral networks that ultimately facilitate timely and efficient referrals [48]. Nonacademic centers can emulate this by working to establish referral networks outside of their institution if these services are not available within their institution. Alternatively, the use of in-person or remote telemedicine patient navigators dedicated to providing patients with FP information and referrals has shown great promise [49]. Another potentially promising intervention is automated referral reminders integrated into the electronic medical record, as this can help to standardize referral patterns and at the very least remind providers to discuss fertility in the context of cancer treatment. An additional benefit of utilizing dedicated patient navigators to assist with referrals and automated referral systems integrated into the electronic medical record is time efficiency, as providers cite time constraints as a substantial barrier to making patient referrals. In a recent qualitative study assessing systemic barriers impacting the decision to pursue FP among reproductive-aged women diagnosed with breast cancer before beginning cancer treatment, time concerns related to independently coordinating referral to a fertility specialist were commonly cited [50]. While automated referral interventions have not been well studied in the FP population, automated referrals have been shown to improve efficiency in other healthcare settings [51]. These time-saving interventions may harness the most potential for addressing disparities in REI referral. Furthermore, as was noted with patient counseling, both patient and provider characteristics appear to influence rates for FP referral. Lastly, female physicians and gynecology oncologists are more likely to refer patients of childbearing age to a specialist. Additionally, patients who ask about fertility in the context of a cancer diagnosis are more likely to be referred to a fertility specialist [29]. This again highlights the need for universal provider bias training.

In addition, after the point of specialist referral, additional attrition occurs at the level of ART access and utilization. This is thought to occur in part for financial reasons due to the cost associated with FP and lack of insurance coverage, even for FP in the setting of a cancer diagnosis. Certainly, while improved insurance coverage could be impactful for patients, previous studies show this would be unlikely to completely address and resolve disparities in FP access and utilization [52,53,54,55]. Therefore, this is likely only part of the problem, requiring us to look beyond insurance mandates. Efforts to reduce patient burden when accessing FP are prudent. Another promising intervention that could ameliorate access barriers for patients interested in FP is the use of telehealth. Such interventions have been highly successful for FP counseling in males with cystic fibrosis, for example [56]. Additionally, among infertility patients who pursue ART to conceive, there is no association between initial visit type and ongoing pregnancy as a result of non-IVF or IVF treatment [57]. Furthermore, telehealth saves time and effort for both patients and providers, and importantly, patients have no preference for in-person or telehealth follow-up for fertility visits [58]. While more research is needed to evaluate the impact of telehealth for counseling, treatment, and outcomes for patients pursuing FP in the setting of a cancer diagnosis, this represents an area of untapped potential. Lastly, to circumvent the mental and physical demands of pursuing specialist care, consideration should be given to bringing services to patients, such as to federally qualified health centers. Though this would require large-scale redistributions of funding, an example of a more immediately feasible option would be to “host” an REI physician after a referral network has been established, if volume allows. This has potential to minimize the need for patient travel, improve no-show rates, and help overall in improving access to FP services for patients planning to undergo cancer treatment.

A recent study by Peipert et al. used geospatial modeling to assess the distribution of oncofertility centers in the United States and found that 3.63 million reproductive-aged females lack access to oncofertility services [59]. It is noted that states with FP mandates have the highest rates of eligible female patients with geographic access [59]. While this finding underscores the importance of insurance coverage for FP, it also corroborates the idea that insurance mandates alone are insufficient to resolve issues of access to FP services. Currently, 18 states have passed FP mandates. However, Achalu et al. revealed that coverage variability introduces additional barriers to qualify for coverage [60]. Inconsistencies exist at multiple levels, including which clinical conditions qualify for FP services, services covered, and the time period for coverage [60,61]. Additionally, these mandates are only applicable to commercial insurance plans. Therefore, patients with publicly funded insurance, including Medicaid, are not eligible for this coverage. Importantly, >50% of racial and ethnic minority patients are covered by Medicaid, and thus such patients have a higher burden of uncovered FP services. A 2022 study completed at a single institution focused on utilization of elective FP in the setting of insurance coverage offered through an employer benefit showed that people were more likely to utilize these services if they were covered [62]. Of note, however, that study excluded patients who were undergoing FP for medical reasons, including a need for gonadotoxic therapy, and thus these conclusions may not be fully extrapolatable to our population of interest. As such, there is a need for studies specifically investigating how state mandates affect FP rates for patients diagnosed with cancer.

Overall, more studies are needed to further explore the impact and etiologies of racial and ethnic disparities in FP for patients diagnosed with cancer to properly address these gaps. Additionally, the few studies focused on this topic are often limited by study design, including small samples or completion at a single institution or site. Future studies should focus on robust study design to allow for maximal extrapolation. The compounding disparities for patients with a new cancer diagnosis from racial and ethnic minority groups seeking FP are highly concerning, as these patients are at the periphery of already marginalized groups. The above highlighted timepoints represent crucial areas where efforts could be concentrated to address disparities in FP for patients diagnosed with cancer (Table 1). Still, ongoing research is needed to further understand the specific factors contributing to such disparities and investigate which interventions are most effective at bridging these gaps.

## 6. Future Directions

The body of literature assessing racial and ethnic disparities in women diagnosed with cancer in the US is relatively sparse. More robust contemporary research studies, specifically with populations representative of the US population, are warranted to better understand current racial and ethnic disparities in FP for women diagnosed with cancer in the US. Additionally, studies focusing on the outcomes of FP services would allow for a more complete evaluation of this topic and to further investigate which interventions have the most potential to being effective in rectifying this issue.

## 7. Conclusions

Numerous studies reveal that racial and ethnic disparities in FP for women with cancer in the US are prevalent. These disparities occur at important time points, including the time of diagnosis, when the effects of cancer treatment on fertility should be discussed, to the point of referral to a REI specialist for discussion of options, to the time point of accessing and utilizing FP services. These multiple bottleneck events compound upon themselves, leaving patients from racial and ethnic minority groups with relatively less access to FP services. A multifaceted approach to this problem is crucially needed to minimize such disparities in women’s oncological healthcare.

## Figures and Tables

**Table 1:** Three main identifiable systemic bottlenecks contributing to racial and ethnic disparities in FP for women with cancer in the United States and proposed solutions.

Main Identifiable Systemic Bottlenecks	Suggested Solutions
Delayed counseling or lack of counseling regarding the reproductive consequences of cancer treatment	Incorporating FP into curricula for oncologists in training Increasing provider education and resources regarding the impact of cancer treatment on reproductive health and FP options Use of patient informational tools and decisions aids Universal provider implicit bias training to address discrepant counseling based on sociodemographic characteristics
Suboptimal referral rates	Continue to build and develop oncology and REI referral networks Utilizing patient navigators for patient education and referrals Integrated electronic medical record generated automated referrals or referral suggestions/reminders to standardize referrals and reduce bias Universal provider implicit bias training to address discrepant referral patterns based on sociodemographic characteristics Institute “opt-out” referrals for women of reproductive age for FP
Reduced rates of ART access and utilization in underrepresented groups	Increasing efforts to bring fertility counseling and services to patients Increasing efforts for patients to understand what insurance coverage and benefits they currently have Improving funding and services for federally qualified health centers (FQHCs) Expanding comprehensive insurance mandates as well as Medicaid to cover FP care/treatment Improve access to REI providers via utilization of telehealth

## Data Availability

No new data were created or analyzed in this study. Data sharing is not applicable to this article.
